# Confronting plastic pollution to protect environmental and public health

**DOI:** 10.1371/journal.pbio.3001131

**Published:** 2021-03-30

**Authors:** Liza Gross, Judith Enck

**Affiliations:** 1 Public Library of Science, San Francisco, California, United States of America; 2 Center for the Advancement of Public Action, Bennington College; Beyond Plastics, Bennington, Vermont, United States of America

## Abstract

A new collection of evidence-based commentaries explores critical challenges facing scientists and policymakers working to address the potential environmental and health harms of microplastics. The commentaries reveal a pressing need to develop robust methods to detect, evaluate, and mitigate the impacts of this emerging contaminant, most recently found in human placentas.

The explosive production of affordable plastic goods during the 1950s ushered in an era of disposable living, fueled by an addiction to convenience and consumerism, that has created one of the world’s most vexing pollution problems. Plastic, for all its uses, has left a trail of debris from the deepest ocean trenches to the remotest polar reaches. Plastic pollutes throughout its life cycle, from its beginnings as a by-product of greenhouse gas-emitting oil and natural gas refining to its degradation-resistant end as increasingly fragmented shards of micro-and nanoplastics in atmospheric currents, alpine snow, estuaries, lakes, oceans, and soils. Researchers are finding microplastics in the gut or tissue of nearly every living thing they examine, including the placentas of unborn children.

The first sign of this burgeoning crisis came nearly half a century ago, when marine biologists first spotted tiny plastic pellets stuck to tiny marine organisms and seaweed in the North Atlantic’s Sargasso Sea. Describing their discovery in 1972, the scientists predicted, presciently, that “increasing production of plastics, combined with present waste disposal practices, will probably lead to greater concentrations on the sea surface” [1].

Researchers have struggled to keep tabs on plastic production and waste ever since. The first global assessment of mass-produced plastics, reported in 2017, estimated that manufacturers had produced 8,300 million metric tons of virgin plastics, creating 6,300 million metric tons of plastic waste—with only 9% recycled, 12% incinerated, and the rest either piling up in landfills or entering the environment [2].

Some 15 million metric tons of plastic enters the oceans every year [3], choking marine mammals, invading the guts of fish and seabirds, and posing unknown risks to the animals, and people, who eat them. Plastics release toxic chemicals added during manufacturing as they splinter into smaller and smaller fragments, with half-lives ranging from 58 to 1,200 years [4]. Persistent organic pollutants have a high affinity for plastic particles, which glom on to these contaminants as do pathogens in the ocean, presenting additional risks to marine life and the food web. Scientists once viewed freshwater lakes and rivers as primarily conduits for plastic, delivering trash from land to the sea, but now realize they’re also repositories.

Plastic production increased from 2 million metric tons a year in 1950 to 380 million metric tons by 2015 and is expected to double by 2050 [2]. Petrochemical companies’ embrace of fracking has exacerbated the crisis by producing large amounts of ethane, a building block for plastic.

Recognizing the scope and urgency of addressing the plastic pollution crisis, *PLOS Biology* is publishing a special collection of commentaries called “Confronting plastic pollution to protect environmental and public health.”

In commissioning the collection, we aimed to illuminate critical questions about microplastics’ effects on environmental and human health and explore current challenges in addressing those questions. The collection features three evidence-based commentaries that address gaps in understanding while flagging research priorities for improving methods to detect, evaluate, and mitigate threats associated with this emerging contaminant.

Environmental ecotoxicologist Scott Coffin and colleagues address recent government efforts to assess and reduce deleterious effects of microplastics, which challenge traditional risk-based regulatory frameworks due to their particle properties, diverse composition, and persistence. In their Essay, “Addressing the environmental and health impacts of microplastics requires open collaboration between diverse sectors” [5], the authors use California as a case study to suggest strategies to deal with these uncertainties in designing research, policy, and regulation, drawing on parallels with a similar class of emerging contaminants (per- and polyfluoroalkyl substances).

In “Tackling the toxics in plastics packaging” [6], environmental toxicologist Jane Muncke focuses on a major driver of the global plastic pollution crisis: single-use food packaging. Our throwaway culture has led to the widespread use of plastic packaging for storing, transporting, preparing, and serving food, along with efforts to reduce plastic waste by giving it new life as recycled material. But these efforts ignore evidence that chemicals in plastic migrate from plastic, making harmful chemicals an unintentional part of the human diet. Addressing contamination from food packaging is an urgent public health need that requires integrating all existing knowledge, she argues.

Much early research on microplastics focused on ocean pollution. But the ubiquitous particles appear to be interfering with the very fabric of the soil environment itself, by influencing soil bulk density and the stability of the building blocks of soil structure, argue Matthias Rillig and colleagues in their Essay. Microplastics can affect the carbon cycle in numerous ways, for example, by being carbon themselves and by influencing soil microbial processes, plant growth, or litter decomposition, the authors argue in “Microplastic effects on carbon cycling processes in soils” [7]. They call for “a major concerted effort” to understand the pervasive effects of microplastics on the function of soils and terrestrial ecosystems, a monumental feat given the immense diversity of the particles’ chemistry, aging, size, and shape.

The scope and effects of plastic pollution are too vast to be captured in a few commentaries. Microplastics are everywhere and researchers are just starting to get a handle on how to study the influence of this emerging contaminant on diverse environments and organisms. But as the contributors to this collection make clear, the pervasiveness of microplastics makes them nearly impossible to avoid. And the uncertainty surrounding their potential to harm people, wildlife, and the environment, they show, underscores the urgency of developing robust tools and methods to understand how a material designed to make life easier may be making it increasingly unsustainable.
